# Unmixing noisy co-registered spectrum images of multicomponent nanostructures

**DOI:** 10.1038/s41598-019-55219-2

**Published:** 2019-12-11

**Authors:** Nadi Braidy, Ryan Gosselin

**Affiliations:** 1 Department of Chemical Engineering and Biotechnological Engineering, Université de Sherbrooke. 2500 Boul, de l’Université, Sherbrooke, PQ J1K 2R1 Canada; 2 Institut Interdisciplinaire d’Innovation Technologique (3IT), Sherbrooke, PQ J1K 0A5 Canada

**Keywords:** Nanoscale materials, Techniques and instrumentation

## Abstract

Analytical electron microscopy plays a key role in the development of novel nanomaterials. Electron energy-loss spectroscopy (EELS) and energy-dispersive X-ray spectroscopy (EDX) datasets are typically processed to isolate the background-subtracted elemental signal. Multivariate tools have emerged as powerful methods to blindly map the components, which addresses some of the shortcomings of the traditional methods. Here, we demonstrate the superior performance of a new multivariate optimization method using a challenging EELS and EDX dataset. The dataset was recorded from a spectrum image P-type metal-oxide-semiconductor stack with 7 components exhibiting heavy spectral overlap and a low signal-to-noise ratio. Compared to peak integration, independent component analysis, Baysian Linear Unmixing and Non-negative matrix factorization, the method proposed was the only one to identify the EELS spectra of all 7 components with the corresponding abundance profiles. Using the abundance of each component, it was possible to retrieve the EDX spectra of all the components, which were otherwise impossible to isolate, regardless of the method used. We expect that this robust method will bring a significant improvement for the chemical analysis of nanomaterials, especially for weak signals, dose-sensitive specimen or signals suffering heavy spectral overlap.

## Introduction

The recent instrumental progress made for the transmission electron microscope (TEM) enabled an improved sensitivity with greater spatial resolution^1^. Thanks to electron probes with higher current density and brightness^2^, energy-dispersive X-ray spectroscopy (EDX) and electron energy-loss spectroscopy (EELS) chemical maps with greater sensitivities can now be routinely collected from nanomaterials with atomic resolution^3^. The challenge of analytical electron microscopy is now to obtain meaningful interpretation of the signal with a minimal electron beam dose^4^. Smaller dose reduces carbon contamination buildup and electron beam damage of sensitive materials while minimizing the total specimen drift during the analysis. In addition, given the high demand and the elevated operation costs of TEM facilities, there is a strong incentive to reduce the beam time and the cost of the analysis.

Spectral imaging is a useful tool to draw chemical maps from multicomponent samples based on compound-specific spectral signatures. The challenge is to separate a large number of spectra into a meaningful number of compounds and to compute their abundance within a region of interest. The complexity of this step can be exacerbated by several factors, especially in the framework of a low dose acquisition. First, the blind source separation (BSS) problem, in which the number and the nature of the sample are unknown, leads to an absence of reference compounds^5^. Second, the presence of heavily overlapping spectral peaks in the spectral signatures hinders the ability to distinguish compounds.

TEM hyperspectral images are typically processed using background removal and peak-integration techniques or linear least-square (LLS) fitting from known references^6^. Obviously, an analytical signal collected with a smaller dose leads to a weaker, and sometimes sparse, signal, which becomes impossible to analyze using conventional windowing techniques. Binning neighboring pixels (energy channels) can increase the signal-to-noise ratio (SNR) to a suitable level but comes at the cost of a reduced spatial (spectral) resolution, which leads to dropped features. In addition, the use of spectral references is not ideal because it can lead to an erroneous estimate of the number and the nature of the species present in the dataset.

A paradigm shift is taking place in the microscopic community in which a wide range of multivariate analysis methods are now being proposed to analyze hyperspectral images^7^. These methods seek to enhance the interpretability of the images by resolving the blind source separation problem in which the number and nature of the compounds, as well as their location in the image, must be determined.

Matrix factorization seeks to decompose an input matrix (e.g. a spectral image) into two or more matrices, called factors, in such a way that the product of these factors approximates the input matrix. Usually, the rank of these factors will be much lower than that of the input matrix, thus creating a low rank approximation, that can in turn assist the analyst interpret the spatial distribution of the components of the region of interest. This is typically achieved by approximating the spectral image ***X*** (*N* × *K*) by 2 factor matrices: spectral signatures of the pure species ***S*** (*J* × *K*) and a chemical map of their abundance in the sample ***A*** (*N* × *J*), where *N* is the number of pixels in the image, *K* is the number of spectral channels and *J* is the number of species identified in the image.

Principal Component Analysis (PCA) is one of the first, and probably best known, multivariate techniques^8^. It is used to compute a projection that maximizes the total variance of the projected data. Such a projection can be used to reduce the dimensionality of spectroscopic data, which can in turn greatly improve interpretability. In transmission electron microscopy (TEM), PCA has often been used to interpret the signals obtained from EDX and EELS^9–11^. Independent Component Analysis (ICA)^12^ computes a linear transformation that minimizes the statistical dependence between its components. In contrast with PCA, ICA not only avoids correlation between second-order statistics but reduces higher-order statistical correlations, in order to render the signals as independent as possible^13,14^. Work using ICA to extract meaningful results from TEM analytical methods can also be found elsewhere^10,15^. Building on this, hyperspectral image analysis using a kernel version of ICA, referred to as KICA, has been presented by Song *et al*.^16^. While all these authors have obtained success with this method in obtaining meaningful results of microscopic data, the use of ICA for hyperspectral image analysis has faced some critiques. ICA retrieves independent sources by considering the input signal as a linear combination of statistically independent signals. As explained by Nascimento and Bioucas Dias^17^ and Villa *et al*.^18^, this assumption does not usually hold for typical hyperspectral images due to the large dimensionality of the data. They conclude that ICA is not suitable for the segmentation of hyperspectral images.

Nonnegative Matrix Factorization (NMF) is another class of matrix factorization methods for which the output matrices are constrained to be nonnegative^19^. Unlike PCA and ICA, these constraints ensure physically meaningful results using NMF (***A*** and ***S***). In TEM analysis, these methods have been used by some authors^20,21^. Multivariate curve resolution (MCR), often performed via alternating least squares (ALS), falls within umbrella of NMF. It however contains additional physically meaningful constraints. These include closure (abundance sum to unity), unimodality (each component has one local maximum), local rank (number of components per pixel), and trilinearity (multiple constraints)^22^. MCR-ALS has often been used on TEM data characterized by high SNR^23,24^ but have been shown to fail for lower SNR^25^. Recently, Baysian linear unmixing (BLU) has also been shown to perform well in spectral image matrix factorization^26,27^. As in MCR, BLU applies nonnegativity constraints to ***A*** and ***S*** as well as closure constraints on ***A***. This is achieved *via* a Bayesian formulation that incorporates constraints directly into the problem.

Recently, our team has developed multivariate curve resolution by log-likelihood maximization (MCR-LLM)^25^. As discussed, MCR-ALS computes the decomposition via the iterative use of classical least-squares regression followed by a correction step to impose a series of constraints. While this method has found much success, the presence of low-count, or low-SNR, data leads to poor quality unmixing. In these cases, the regression steps provide poor estimates of ***A*** and ***S*** which cannot be meaningfully corrected by the constraints, and propagates at each iteration. In our variant of MCR, a least-squares regression step was replaced by a Poisson log-likelihood optimization of the abundance of each component for a given pixel. In considering the Poissonian nature of the data, the algorithm avoids the subsequent corrections that corrupt the response. MCR-LLM has been shown to outperform MCR-ALS for low count and low-SNR data^25,28^.

The objective of this work is to demonstrate that multivariate curve resolution by log-likelihood maximization (MCR-LLM), an algorithm recently developed by our team, outperforms other multivariate methods.

For this work, MCR-LLM is compared to ICA, MCR-ALS, NMF and BLU, all recently proposed algorithms for the analysis spectrum images of complex microstructures. To achieve this, a particularly challenging dataset was chosen. Acquired both in EELS and EDX, the image presents a micro-fabricated sample in which a large number of species are captured by a very small number of low-SNR spectra. Such a challenging dataset is crucial as it cannot be analyzed by traditional methods and exposes the limitations of the multivariate methods.

## Methodology

**Figure 1:**
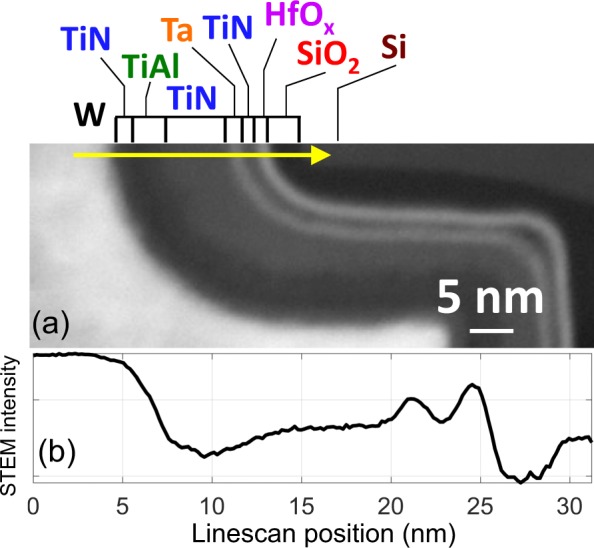
(**a**) High angle annular dark field image of the Ivy bridge. Arrow indicates EELS-EDX linescan trace. (**b**) Logarithm of the STEM intensity profile across linescan. Colored text above the figure correspond to the color code of the phases in Figs. 4 and 5.

A thin (~40 nm) lamellae of an Intel Xeon Ivy Bridge tri-Gate 22 nm process was prepared by focused ion beam (FIB) and lifted out for STEM analysis. The sample presented 9 layers consisting of 7 phases: W, TiN, TiAl, Ta, HfO_x_, SiO_2_ and Si. The analytical data was collected using a ~0.1 nm diameter electron beam scanning transmission electron microscopy (STEM) using an aberration corrected FEI Titan TEM at 300 kV. The region of interest was imaged with a convergence angle of 18 mrad in Gatan STEM annular dark field (Fig. 1: STEM image and profile) using an inner angle of 40 mrad and an outer angle of 80 mrad. EELS and EDX spectrum images were simultaneously acquired on a 31 nm linescan (100 pixels).

EELS spectrum image was collected with a 40 mrad collection angle on a Gatan Image Filter GIF966 set in parallel acquisition of a spectrum using 2048 channels between 1100 eV and 2100 eV. The EDX spectrum image was acquired in parallel using an Oxford detector (0.13 steradians solid angle) over 4096 channels between 0–20 keV. The dwell time per pixel for both acquisitions was set to 4 s. Another EELS dataset was acquired over the region of interest in the 60–1084 eV range to confirm the C, N, Ti and O distribution in the various layers.

**Figure 2:**
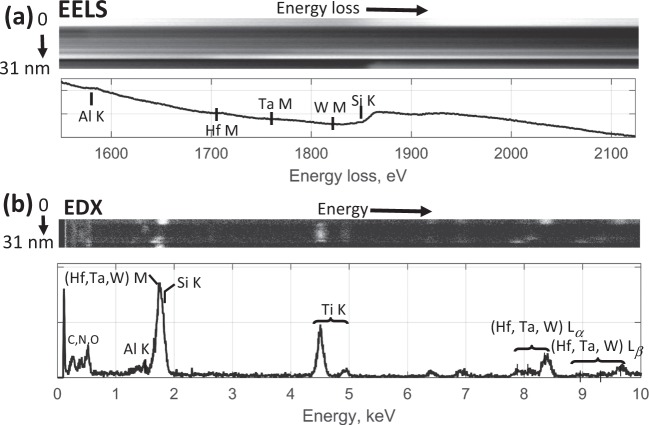
EELS (**a**) and EDX (**b**) representation of the spectrum image dataset and corresponding sum spectra.

Both EELS (Fig. 2a) and EDX (Fig. 2b) spectra exhibit strongly overlapping features and low SNRs. Typical peak integration failed to produce meaningful interpretation whereas linear least-square fits using internal references resulted into noisy elemental profiles that lack internal consistency (Fig. S2).

**Figure 3:**
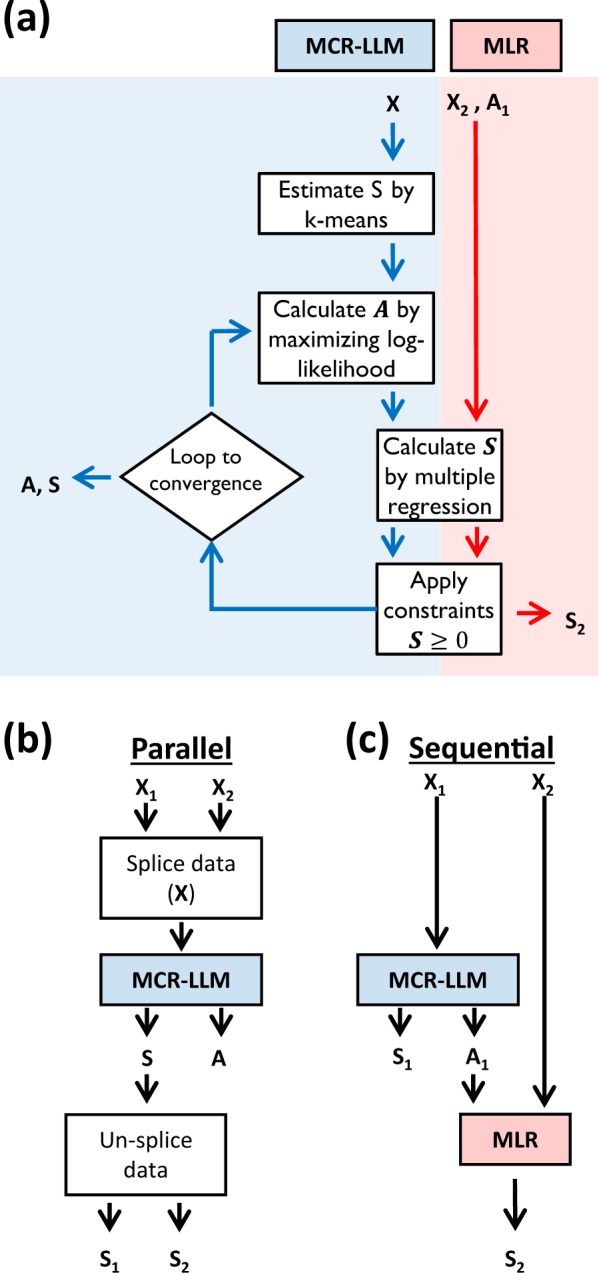
(**a**) Algorithm of the multivariate curve resolution loglikelihood maximization (MCR-LLM) and the multiple linear regression (MLR). Overall strategies for determining the abundance (A) and the spectral endmembers (S): (**b**) parallel and (**c**) sequential computation.

As illustrated in Fig. 3a, MCR-LLM is used to identify the spectral signatures of the endmembers ***S*** (J × K) and their abundance map in the sample ***A*** (N × J) within a spectral image ***X*** (N × K). To do so, k-means clustering produces an initialization set of *J* spectral components. These are used as initial estimates in an iterative process that optimizes the contribution of the spectra via log-likelihood under constraints of closure and non-negativity. The spectra ***S*** are computed using ***X*** and ***A*** via standard multilinear regression and corrected under constraints of non-negativity. The error envelopes of the EELS spectra were computed using a resampling methodology described in the Supplementary Information (S3). These steps are iterated to convergence to obtain the final ***A*** and ***S***. Full details of the method can be found in Lavoie *et al*.^25^. This method is particularly well suited for low SNR spectra for which the Poissonian nature of the data becomes prominent.

The dataset (Fig. 2) was acquired using EELS and EDX acquisition, with both modalities collected simultaneously from the same area on the sample. In such cases, the modalities can be analyzed separately or in combination using either one of the aforementioned algorithms: ICA, MCR-ALS, NMF, BLU and MCR-LLM. None of these algorithms was able to extract meaningful endmembers or abundance maps from the EDX spectrum image taken alone due to its high dimensionality and low SNR. In comparison, it was easier to extract significant endmember spectra and corresponding abundance maps for the EELS spectrum image, which contains a higher SNR.

Nevertheless, the EDX data complements the EELS by providing meaningful insight on the abundance of elements that are not probed in the EELS energy range (1100 eV-2100 eV) such as C, N, O and Ti and by confirming the nature of the identified endmembers.

Here, two strategies were sought to combine both EELS and EDX co-registered dataset to maximize the interpretability and the information quality. First, a parallel analysis of the modalities (Fig. 3b) was carried out in which the EDX (100 × 4096) and EELS (100 × 2048) datasets were spliced into a single matrix (100 × 6144) prior to the blind source separation. The output ***S*** and ***A*** matrices can then be separated to create individual matrices for both modalities (***S***_***i***_ and ***A***_***i***_). Tests show that such a combination of the EDX and EELS yields inferior results to those obtained using EELS alone. The high dimensionality of the EDX data, coupled to its low SNR, rendered the initialization step unreproducible, thus hindering the analysis of EELS alone.

Here, we propose an innovative approach to harness the information of poor data by leveraging a high quality co-registered information. We developed a sequential analysis (Fig. 3c) in which MCR-LLM is first applied to the higher quality data, in this case EELS, to generate the exact endmember signatures ***S***_***1***_ and corresponding abundance maps ***A***_***1***_. Then, using multiple linear regression we leverage the EELS abundance map, ***A***_***1***_, to infer the corresponding lower-quality EDX endmember signatures, ***S***_***2***_.

## Results

**Figure 4:**
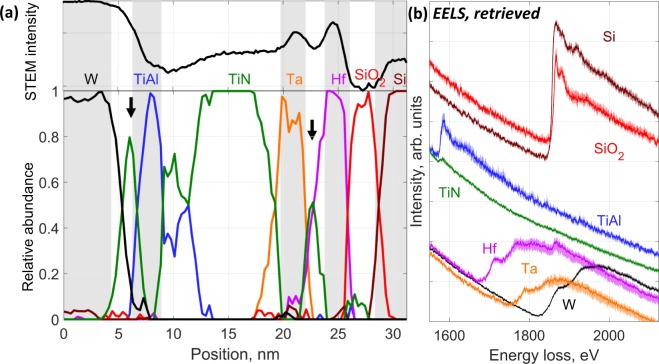
Retrieved abundance profile (**a**) and corresponding EELS spectral endmembers. (**b**) EELS spectra are shown with confidence envelope calculated using a procedure described in the SI (S3). Spectra were scaled and offset for clarity. The logarithm of the STEM-ADF intensity is plotted on top of the image with corresponding shaded areas (Fig. 1) are drawn as a guide. Arrows on abundance map indicate the presence of thin TiN gate layers.

The retrieved abundance (***A***) profiles (Fig. 4a) reveal the presence of spatially resolved pure phases without making a prior assumption on the pixel neighborhood. The position of the phase boundaries matches the ADF contrast of the analyzed region (watermark on Fig. 4a) and the expected position of the various layers composing the P-type metal-oxide-semiconductor (PMOS) gate dielectric. We note that the 1 nm-thick (FWHM ~3 pixel) TiN gates between W and TiAl (5–6 nm) and Ta barrier and Hf dielectric (21–22 nm) are clearly resolved but overlap with neighboring phases due to the finite beam size and the beam broadening across the thickness of the sample. Using the relationship proposed by Gauvin & Rudinsky^29^, and considering a beam size of 0.1 nm, a 40 nm thickness and a convergence angle of 18 mrad, the beam broadening that accounts for 90% of the electrons over the 2 nm Ta layer is estimated to ~3.6 nm. The beam diameter at the bottom of the sample is estimated to ~3.8 nm. The scale of the broadening is consistent with the size of the tails of the Ta component profiles of Fig. 4a.

The method successfully extracted the expected EELS spectra of Ti, Hf, Ta and W based layers (Fig. 4b). The error envelopes of the EELS spectra are tightly bound to the calculated MRC-LLM spectra. The width of the error envelope is thickest for the minor phases: Hf, Ta and SiO_2_. In addition, Si and SiO_2_ could be discriminated, without the use of internal references. The endmember spectra associated to the HfO_x_ layer highlights the presence of Si, which is not visible using least-square fitting (Fig. S2), demonstrating the systematic presence of Si in that layer. We note that the featureless EELS background associated to the Ti-based layers over the 1100–2100 eV energy range (Fig. 4b) could be isolated. In this case, removing the background of the EELS spectra prior to analysis would result in the loss of the Poisson character of the signal and make it difficult to uncover the Ti-based layers and their respective contribution to the EELS signal (in the 1100–2100 eV range).

**Figure 5:**
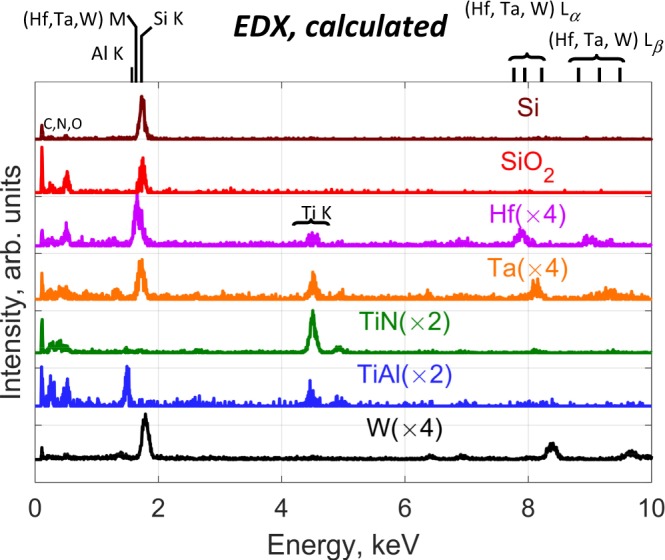
Calculated EDX endmembers using the retrieved EELS dataset. Spectra were scaled and offset for clarity.

As discussed, the MCR-LLM classification schemes fail with the EDX signal alone. Splicing it with the EELS signal did not improve the phase retrieval process. However, it was possible to calculate the EDX endmembers (Fig. 5) from the EELS abundance map and the EDX dataset, following the MLR scheme presented in Fig. 3c. In this case, Si and Al K EDX lines associated to the Si, SiO_2_ and TiAl phases are easily separated from the overlapping M lines of the heavy metals. The heavy metal M and L bundles can be easily discriminated in the EDX endmembers (Fig. 5) with very little or no spectral features from other components. For instance, Ta shows traces of Ti because it is sandwiched between two TiAl layers, which overlap the pure Ta component. The systematic detection of Ti in the Ta and Hf layer was confirmed using an EELS spectrum image recorded in the 60–1084 eV range. O K edge is observed in the HfO_x_, and SiO_2_ layers as an intrinsic part of these phases. The O K peak is also observed as a minor element, probably as surface oxidation, for Ta and TiAl and is detected as trace element for W and TiN.

## Discussion

The results demonstrate the ability of MCR-LLM to blindly extract multiple and true components from small size dataset with low counts. As demonstrated by the error envelopes (Fig. 4b), the method is very robust and facilitates the interpretation of the phase spectral signatures and spatial features. The technique assumes that the data is corrupted with Poisson noise. Therefore, it is important to conduct the operations with the raw dataset, prior to background subtraction or other form of data pre-treatment. The analyzed signal intensity should be positive and consisting of integer counts. In our case, the pixels were corrected for gain-to-gain variations and dark current prior to analysis. Ideally, the data should be recorded without these corrections to retain the true Poissonian nature and added to the spectra of the extracted endmembers. Nevertheless, we have validated that the signal follows a Poisson distribution for the EDX dataset. For the EELS dataset, the variance is ~1.63 × mean (see demonstration in Supplementary Information S4), but given the strong signal of the EELS data, the distribution tends towards a Gaussian. With such a strong signal, any departure from a Poisson distribution will also be Gaussian and should not invalidate the procedure. We expect that MCR-LLM would perform even better with single-electron detectors, which signal is purely Poissonian.

Our method also assumes that the signal scales linearly with the abundance of the components. The EELS signal becomes non-linear in the presence of local thickness variations. The signal is convolved to the low loss of the EEL spectra, which features varie strongly with thickness. In our case, the thickness uniformity was ensured by the uniform FIB cutting. If the high loss is strongly nonlinear, we can use a co-registered low-loss EELS dataset to deconvolve the core-loss signal. However, this deconvolution process would risk altering the Poissonian nature of the dataset. Lastly, we should note that the MCR-LLM does not affect the energy resolution of the signal.

The technique presented herein proposes to extract the spectral components rather than the elemental edges. The standard power law background subtraction and windowing procedures for quantification can be carried out on the retrieved endmembers with much more precision than the traditional pixel-by-pixel analysis.

With a higher SNR, the elements forming the endmembers are more easily studied. It is thus possible to quantify the elemental content with higher confidence and offers a more legible edge fine structure that can be compared against a collection of references. Once the elemental contribution to each endmember is determined, the abundance map can be easily converted to elemental maps *via* sensitivity factors. Using the matrix form outlined in Section 2, consider the elemental composition of each component expressed as matrix a ***E***(J × M) with J components and M elements. The matrix ***C*** (N × M) obtained by the matrix product (***A E***) would thus represent the elemental composition for each pixel.

We stress that we have purposely chosen a challenging dataset with a low number of pixels (100) with strongly overlapping signal to demonstrate the robustness of the method. Obviously, using a greater number of pixels over the region of interest, a longer dwell time or focusing on the 100–1000 eV energy loss could have facilitated the analysis. Note that the method does not consider neighboring pixels and is thus independent on the dimensionality of the array. Therefore, the method can be directly applied to 2D, 3D hyperspectral datasets and even for chronospectroscopy datasets. Larger datasets are longer to process but contain more information. In this case, the endmembers spatial distribution and spectral signature are easier to retrieve. The performance of the retrieval algorithms is measured in the ability to extract meaningful information from poor quality datasets rather than from the capacity to process large datasets.

The proposed method outperforms a series of algorithms that were recently used for data analysis (Figs. S5–S7) referred to in the Introduction. These methods are inappropriate for spectral imaging and fail for dataset with a limited number of low-count pixels. PCA generates physically meaningless loadings together with positive and negative scores that cannot be interpreted as spectra. As alluded to in the Introduction, ICA assumes that the dataset is composed of independent variables, which is not the case for spectrum-image datasets given than the number of channels far exceeds to the number of components. As for K-ICA, it transforms the data using a user-defined and subjective kernel. K-ICA (Fig. S5) and multiple variants of NMF (Fig. S6) failed to provide meaningful and realistic profiles for the EELS dataset.

When dealing with low count data, MCR-ALS clips negative abundances to 0, which skews the contribution of the other components during the normalization step and derails subsequent iterations. Our algorithm circumvents the normalization step by replacing it with a loglikelihood maximization operation. N-FIND-R-initialized BLU algorithm shows well separated spectral signatures (Fig. S7), except for the SiO_2_ component, although the abundance maps are rather noisy and some components are mistakenly positioned (SiO_2_, W and TiN). Because NFIND-R is based on a subset of observations, the initialization step can be very sensitive to outliers, especially for a low number of observations.

All the methods fail when analyzing the EDX dataset, including MCR-LLM. In this case, the results lack robustness because of random aspect brought by the k-means initialization. Spectral binning improved the SNR and the overall results but with the obvious drawback of reduced spectral features. MCR-LLM converged to incorrect EDX spectra features and maps independently of the spectral binning. For these reasons, splicing the EDX dataset to the EELS hindered the retrieval process. In this particular case, the EELS abundance map helped retrieve meaningful information contained in the EDX dataset that complemented the EELS information. If the EDX dataset was acquired with a higher SNR, it could likely have been jointly analyzed with the EELS dataset to improve the overall results (Fig. 3b).

## Conclusion

The methods for the analysis of spectrum images in microscopy are undergoing a paradigm shift for which the background-subtracted windowing methods are replaced by multivariate methods to blindly retrieve the spectral endmembers and corresponding abundance maps. In this context, we propose a new method, MCR-LLM, which is particularly well suited for low-count datasets with multiple sources or severe spectral overlap. We demonstrated the applicability and the limit of the algorithm for a co-registered EELS and EDX datasets recorded from Ivy-bridge stack made of 7 different components exhibiting strong superposition of the various elemental signals.

Using MCR-LLM, it was possible to retrieve the EELS spectral signature and abundance map of the 7 components that reproduces the known architecture of the stack. The EDX SNR was too low for MCR-LLM to process but can be generated using a multilinear regression using the retrieved EELS abundance. The traditional windowing methods fail to produce meaningful results and suffer from numerous artefacts arising from the strong overlap of the signals and the weak SNR. Other multi-linear approaches, including ICA and NMF, provide erroneous profiles and spectra, although BLU offers acceptable, but imperfect separation.

MCR-LLM allows high quality spectra of the components to be isolated. This method, along with several other multivariate techniques, should be part of the routine analysis toolbox of spectrum image datasets. These tools will allow the analyst to achieve a much more detailed and comprehensive analysis of spectrum images. In addition to enabling the extraction of meaningful spectral components, this method makes it possible to carry out a single analysis of several co-registered datasets. A more powerful tool for data processing also enables the interpretation of lower SNR compared to traditional methods, which limits the necessity of sacrificing spatial resolution to improve the analysis for new or novel materials. The ability of improved data analysis also translates into lower beam dose requirements to achieve a given level of interpretation. This method would be particularly insightful for EELS datasets recorded using a spectrometer fitted with a single-electron camera. Work is under way to consider non-linear phenomena associated to changes in thickness, known to influence the EELS post-edge structures and the EDX background.

## Supplementary Information

Supplementary Information

## Data Availability

The datasets generated during and/or analyzed during the current study are available through a PyPi repository, pypi.org/project/MCRLLM/.
