# Supplementary material for: Unmixing noisy co-registered spectrum images of multicomponent nanostructures
Source: Sci Rep. 2019 Dec 11;9:18797. doi: 10.1038/s41598-019-55219-2 (PMC6906416; doi:10.1038/s41598-019-55219-2)
Supplement: Supplementary file 1 — Supplementary Information [file 41598_2019_55219_MOESM1_ESM.docx]

Supplementary information to “Unmixing noisy co-registered spectrum images of multicomponent nanostructures”

**Nadi Braidy*, Ryan Gosselin**

Department of Chemical Engineering and Biotechnological Engineering, Université de Sherbrooke. 2500 Boul. de l’Université. Sherbrooke, PQ J1K 2R1, Canada

Institut Interdisciplinaire d'Innovation Technologique (3IT), Sherbrooke PQ J1K 0A5, Canada

The data contained in this Supplementary information supports the discussion of paper entitled “Enhanced interpretability of nanostructure spectrum images using novel multivariate methods”. The following presents results of “traditional” data analyses (background-subtracted windowing, S1, and linear least-square fitting, S2) together with the results of various other latent variable algorithms: Independent component analysis (ICA, S3) Non-negative matrix factorization (NMF, S4), and Baysian linear unmixing (BLU, S5).

1. Background subtracted windowing

Figure S1. Excerpts of one EDX (a) and EEL (b) spectra showing the high signal-to-noise ratio with the overlapping characteristic features. EDX (c) and EELS (d) profiles of the background-subtracted signal.

Figure S1 shows the line profiles of the background-subtracted analysis typically carried out by commercial software. Figures S1a and S1b show the difficulties associated to those methods when dealing with low signal-to-noise ratio (SNR) and overlapping features in a) EDX and b) EELS data. With sparse data, the slope of the linear EDX background varies significantly depending on the number of pixels used for the extrapolation (Figure S1a). For EELS, the number of pixels used for the background extrapolation is limited by the energy-loss near edge structure (ELNES) of the previous edge (Figure S1b). In both cases, the imprecise background extrapolation and the limited number of counts cause a large uncertainty on the background-subtracted signal. In addition, conventional methods are user-dependent and contain several arbitrary choices that are based on experience, although software to optimize the parameters are available (e.g. “EELS Advisor”, available as a package in Gatan Microscopy Suite Software, Digital Micrograph, from Gatan, Inc). For instance, the user decides the choice of edges or the peaks to extract, the position and the width of background and the signal integration window.

The processed EDX and EELS signals using this background-subtracted method are shown in Figures S1c and S1d, respectively. From these profiles, several artefacts become apparent and result from the low SNR and the signal overlap. All profiles are noisy and include negative counts resulting from the extrapolated background being locally larger than the signal. Of note, confusion arise between (Si, SiO_2_) and W for both EELS and EDX because of the overlapping features of these two peaks. The same phenomena is responsible of the apparent presence of Ta within the Hf-rich layer. The relevant integration ranges of the characteristic EDX and EELS used to generate Figures S1c and S1d profiles are reported in Table S1.

Table S1. Background-subtracted integration ranges used to generate the EDX and EELS profiles in Figs. S1c) and S1d).

| Element | EDX | EELS |
| --- | --- | --- |
| Al | $K_{\alpha+\beta}$: 1450-1550 eV | $K$ : 1571.0-1611.0 eV |
| Si | $K_{\alpha+\beta}$: 1590-1905 eV | $K$ : 1853.0-1880.5 eV |
| Ti | $K_{\alpha+\beta}$: 4345-5115 eV | N/A |
| Hf | $L_{\alpha}$: 7850-7950 eV and  $L_{\beta}$: 8855-9115 eV | $M$ :1696.5-1736.5 eV |
| Ta | $L_{\alpha}$: 8060-8120 eV and  $L_{\beta}$: 9255-9435 eV | $M$ :1768.0-1808.0 eV |
| W | $L_{\alpha}$: 9565-9765 eV,  $L_{\beta}$: 9605-9785 eV and  $L_{\gamma}$: 11125-11385 eV | $M$ :1867.5-1907.5 eV |

1. Linear least-square fitting

EDX and EELS internal references were generated from the sum of three neighboring pixels from each of the seven distinct regions of the Ivy Bridge Tri-Gate. Each EDX and EELS spectrum was then linearly fit to the references to extract a coefficient. The coefficients were normalized to unity and plotted against the position to generate profiles of abundance (Figure S2).

Figure S2. Linear least square (LSQ) fit of (a) EDX and (b) EELS dataset with the corresponding chi square statistic (bottom panel).

The EDX profiles are noisier than the EELS because of its lower SNR. Both signals contain regions of negative relative abundance and exceed unity in others. The regions from which the internal references were extracted correspond to the lower $\chi^{2}$ ridges, in Figure S2b. Here, the resulting profiles appear acceptable but require that high quality spectra of endmembers exist and are identified within the dataset or acquired in identical conditions. LSQ fails to identify other independent species in the dataset but can be suspected in presence of a higher value of the $\chi^{2}$, or goodness-of-fit, which indicates that the references were not sufficient to explain the data.

1. Profile and spectra envelope computation

The error envelopes were created using a resampling procedure in which MCR-LLM models were run on random subsets of the data. We followed a standard procedure in the field of multivariate statistics in order to create confidence intervals on the spectra to illustrate the accuracy of the procedure. Typical results are presented in Figure S3 in which 70 of the 100 pixels were used in each subset MCR-LLM analysis (100 subsets were computed in all). Figure S4 illustrates the 100 raw pixels (grey disks) alongside the full MCR-LLM (7 black crosses) and the 100 MCR-LLM subset analyses (700 spectra: 100 analyses x 7 components). These were clustered using k-means into 7 phases. Outlying spectra within each of these clusters were removed and are shown with a color code consistent with Figure 2. We defined outlying spectra as any spectra falling outside the 95% confidence intervals following the standard procedure^S1^: we applied Hotelling’s T^2^ distance within the PCA subspace and the squared prediction error orthogonal to the PCA subspace. Two principal components were chosen for all PCA analyses based on the estimated effective rank to exclude the component associated to noise.

Figure S3. Score plot of the complete EELS dataset (grey disks). Color dots represent the resampled data that were used to estimate the error envelopes of Fig. 2b. See Section S4 for details.

1. Poisson nature of the dataset

The Poisson nature of the dataset can be demonstrated by plotting the variance against the mean of a uniform subset of the data (Figure S4). If the variance is equal to the mean, then the signal has a Poisson nature. The variance and the mean of the datasets were estimated using a moving average of a background subtracted signal. This strategy was adopted as no spectra acquired at differing doses over a uniform area were available. Therefore, the background was locally modeled over 41 channels using a 2^nd^ order polynomial fit. Then, the signal mean and variance of the background subtracted signal were both computed over the same 41 channels. While a modeling window of 41 pixels was used, the numerical value and overall strategy for subtracting the background was found to have little effect on the results.

With a regression of 0.97, the distribution of the EDX dataset is Poisson. However, the variance of the EELS dataset is larger than the mean by a factor of ~1.63, which signals a departure from the Poisson distribution. As the EELS signal has a strong signal (6200 counts on average), its Poisson distribution approaches that of a Gaussian distribution. A departure for a strictly Poisson distribution at such a large number of counts is also expected to be Gaussian in nature and therefore should not invalidate the procedure.

Figure S4. Variance against the mean of the intensity compiled from a subset of the spectrum image of the a) EELS and b) EDX datasets with corresponding linear regression (red line). A 1:1 slope indicates a signal that has a perfect Poisson noise.

1. Independent component analysis (ICA)

The data was processed using Independent component analysis (ICA). Here, Figure S5 was generated using the FAST ICA algorithm imported from Scikit-learn ^S2^ and processed using Python to generate a blind separation with 7 sources. The Si, W and Hf EELS spectra were successfully retrieved using FAST ICA but some rotational ambiguity persists with the SiO_2_, Ta, TiN and TiAl components. The abundance trace contains large regions of negative abundances, which arise because of the absence of constraints during the iterations. Transforming the data using a radial basis, Hermite and Laplace functions prior to the data processing did not improve the quality of the analysis.

Figure S5. (a) Abundance map and corresponding (b) EELS spectra retrieved using FAST ICA algorithm.

1. Non-negative matrix factorization (NMF)

Non-negative matrix factorization (NMF) was also used for the data analysis. Figure S6 shows the results of the NMF using the “Multiplicative Update Rule” (MUR) ^S3^ . Different responses were obtained by re-running the script several times. Using this technique, Si, Hf, W, Ta edges were isolated but without their respective power law background. All the abundance traces are positive, thanks to the non-negative constraint but some confusion arise between the TiAl, TiN and SiO_2_ components. The 19 variants of NMF referred to in Li et al. ^S3^ did not improve the results shown in Figure S3. Another variant to NMF, Multivariate curve resolution alternating least square (MCR-ALS) ^S4^ did not offer an improvement compared to NMF-MUR shown here.

Figure S6. (a) Abundance map and corresponding (b) EELS spectra retrieved using NMF-MUR algorithm.

1. Baysian linear unmixing (BLU)

Of all the alternative algorithms tried, Baysian Linear Unmixing ^S5^ (with N-finder initialization ^S6^) computed abundance maps and endmember spectra closest to the expected response (Fig. S7). The Hf, Ta and W M lines are clearly resolved, the TiN EELS featureless signature is well captured although the SiO_2_ endmember spectra is noisy. The abundance map shows a component distribution mostly consistent with the expected one but some shortcomings are present. In particular, the abundance profiles do not saturate to 1 as expected and a few regions exhibit false positives: SiO_2_, W and TiN appear in regions 7-12 nm, 21-23 nm and 26-29 nm, respectively.

Figure S7. (a) Abundance map and corresponding (b) EELS spectra retrieved using BLU with N-FIND-R algorithm.

1. Bibliography

S1. Ketelaere, B. De, Hubert, M. & Schmitt, E. Overview of PCA-Based Statistical Process-Monitoring Methods for Time-Dependent, High-Dimensional Data. *J. Qual. Technol.* **47**, 318–335 (2015).

S2. Pedregosa, F. *et al.* Scikit-learn: Machine Learning in Python. *J. Mach. Learn. Res.* **12**, 2825–2830 (2011).

S3. Li, Y. & Ngom, A. The non-negative matrix factorization toolbox for biological data mining. *Source Code Biol. Med.* **8**, 1–15 (2013).

S4. Tauler, R., Kowalski, B. & Fleming, S. Multivariate Curve Resolution Applied to Spectral Data from Multiple Runs of an Industrial Process. *Anal. Chem.* **65**, 2040–2047 (1993).

S5. Dobigeon, N., Moussaoui, S., Coulon, M., Tourneret, J. Y. & Hero, A. O. Joint Bayesian endmember extraction and linear unmixing for hyperspectral imagery. *IEEE Trans. Signal Process.* **57**, 4355–4368 (2009).

S6. Winter, M. E. N-FINDR: an algorithm for fast autonomous spectral end-member determination in hyperspectral data. in *Proc.SPIE* **3753**, (1999).
